# Two coupled circadian oscillations regulate *Bmal1-ELuc* and *Per2-SLR2* expression in the mouse suprachiasmatic nucleus

**DOI:** 10.1038/s41598-018-32516-w

**Published:** 2018-10-03

**Authors:** Shinya Nishide, Sato Honma, Ken-ichi Honma

**Affiliations:** 1 Department of Physiology, Faculty of Medicine, Hokkaido University, Hokkaido, Japan; 2 Department of Occupational Therapy, School of Rehabilitation Science, Health Science University of Hokkaido, Hokkaido, Japan; 3 Research and Education Center for Brain Science, Hokkaido University, Hokkaido, Japan; 4 Study Group for Monitoring of Brain Functions, Hokkaido University Graduate School of Medicine, Hokkaido, Japan

## Abstract

Circadian rhythms in clock genes, *Bmal1* and *Per2* expression were monitored simultaneously in the cultured slice of mouse suprachiasmatic nucleus (SCN) by dual bioluminescent reporters. In the neonatal SCN, the phase-relation between the *Bmal1* and *Per2* rhythms were significantly changed during culture. Medium exchange produced phase-dependent phase shifts (PRCm) in the *Bmal1* rhythms, but not in the *Per2* rhythms. As a result, the two circadian rhythms were temporally dissociated after medium exchange. In the adult SCN, the phase-relation between the two rhythms was kept constant during culture at least up to 20 cycles. The amplitude of PRCm in the adult SCN was significantly attenuated in the *Bmal1* rhythm, whereas a PRCm was developed in the *Per2* rhythm. The circadian period was not systematically affected by medium exchange in either of rhythms, regardless of whether it was in the neonatal or the adult SCN. Tetrodotoxin, a sodium channel blocker, enhanced the phase-response in both rhythms but abolished the phase-dependency. In addition, tetrodotoxin lengthened the circadian period independent of the phase of administration. Thus, the *Bmal1* and *Per2* rhythms in the SCN are dissociable and likely regulated by distinct circadian oscillators. *Bmal1* is the component of a *Bmal1*/REV-ERBa/ROR loop and *Per2* a *Per*/*Cry*/BMAL1/CLOCK loop. Both loops could be molecular mechanisms of the two circadian oscillators that are coupled through the protein product of *Bmal1*. The coupling strength between the two oscillations depends on developmental stages.

## Introduction

The circadian pacemaker in mammals is located in the hypothalamic suprachiasmatic nucleus (SCN). A unilateral SCN consists of ca. 10,000 neurons, the most of which show circadian rhythms in neuronal activity, clock gene expression and intracellular Ca^2+^ concentration^1–3^. According to a current hypothesis, the cellular circadian rhythm is generated by a molecular auto-feedback loop in which 4 clock gene families and their protein products are involved^4^. Expression of clock gene *Per*(s) and *Cry*(s) are activated by a heterodimer of clock gene products BMAL1 and CLOCK. The gene products of *Per*(s) and *Cry*(s) in turn suppress the transactivation of BMAL1/CLOCK (core loop). On the other hand, *Bmal1* expression is activated by RORs and repressed by REV-ERBα^5,6^. Expression of Rev-Erbα is activated by BMAL1 and CLOCK through E-box and Rev-Erbα/ROR response element^5,7^. Thus, the circadian rhythm in *Bmal1* expression is also regulated by an auto-feedback loop (*Bmal1* loop) which interlocks with the core loop. Theoretical evidence has been provided for a potential role of the Bmal1 loop as an independent oscillator^8^. The phase-relation of two loops are represented by the circadian peaks of *Per2* (core loop) and *Bmal1* (*Bmal1* loop) expression, which are almost antiphasic in a steady state condition.

The SCN starts to oscillate from the fetal period in mice^9–11^. The fetal and neonatal circadian rhythms in the SCN are entrained by non-photic signals, known as maternal entrainment^12,13^. Maternal entrainment is replaced by light entrainment at around the postnatal day 5–7 in rats^14^. The mechanism of maternal entrainment and the switch from non-photic to photic entrainment are not well understood. There is a line of evidence that the SCN neural networks in the neonatal period and adulthood are different. For example, D_1_-dopamine receptors activated c-fos expression in the fetal but not adult SCN in rats^15,16^. Circadian rhythms in vasoactive intestinal peptide (VIP) in the SCN, a neuropeptide critical for coherent circadian oscillation, show an endogenous nature in neonatal but lose it in adult rats^17^. *Cry1* and *Cry2* are not necessary for the circadian oscillation in the neonatal mouse SCN, whereas they are crucial for the adult^18^. Previously, we found that medium exchange induced phase-dependent phase-shifts of circadian *Bmal1* rhythm in the cultured SCN slice of neonatal mice but not of the adult^19^. Thus, the circadian rhythms in the SCN make developmentally different responses to environmental perturbations, which could be ascribed to the SCN neural network^20,21^ and responsible for maternal entrainment in the early life and light entrainment afterwards.

In the present study, taking advantage of a dual bioluminescence reporter system for *Bmal1* and *Per2* expressions^22,23^, circadian rhythms of both gene expressions were simultaneously determined in the cultured SCN slices from neonatal and adult mice. ELuc, a reporter of *Bmal1* expression, emits a shorter wavelength and SLR2, a reporter of *Per2* expression, emits a longer wavelength (Supplementary Fig. 1). The two reporter signals were successfully separated by using a 620 nm long pass filter. Here, we report internal dissociation of circadian *Bmal1* and *Per2* rhythms and their differential responses to external perturbation, suggesting distinct circadian oscillators involved in these rhythms. The responsiveness of these circadian rhythms depends on the developmental stage.

## Results

### Circadian rhythms of Bmal1 and Per2 expression in the cultured SCN slice

Coronal brain slices of 300 μm thickness including the middle part of SCN were obtained from neonatal (6 days old) and adult (2–5 months old) mice of both sexes carrying dual bioluminescence reporters. A bilateral SCN was dissected from the slice with a surgical knife and explanted on a culture membrane for a long term culture. *Bmal1-ELuc* and *Per2-SLR2* expression were measured simultaneously at 20 min intervals. The reporter signals were successfully separated by a long-pass filter (Supplementary Fig. 2). The transmission coefficient was stable and independent of the time of day and the intensity of bioluminescence in the range measured in the present study (Supplementary Fig. 1).

**Figure 1:**
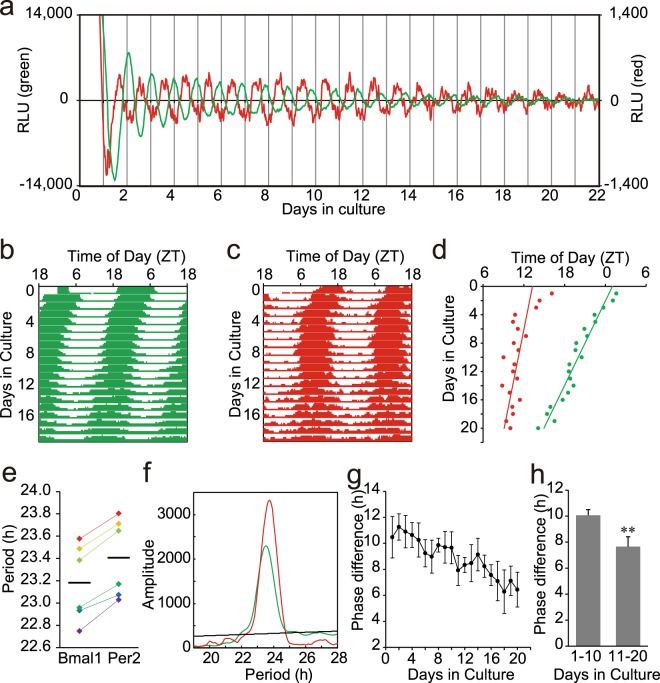
Circadian rhythms in the neonatal SCN slices of *Bmal1-Eluc:Per2-SLR2* mice (**a**) Sequential records of circadian *Bmal1* (green) and *Per2* (red) rhythm in a neonatal SCN slice. Original records were smoothed by a 5 point (80 minutes) moving average method and detrended by a 24 hour moving subtraction method. The abscissa indicates the day in culture and the ordinate the relative intensity of bioluminescence (right, *Bmal1*; left, *Per2*). Day 0 means the day of slice preparation. (**b**,**c**) A double plotted circadian *Bmal1* (**b**) and *Per2* (**c**) rhythm in a raster format. Bioluminescence data of each day were indicated as a percentile to the daily mean. (**d**) Successive plots of circadian peak phases (*Bmal1*, green; *Per2*, red) and regressions lines fitted to them in the same SCN slice as used in (**a**). (**e**) Circadian periods of individual *Bmal1* and *Per2* rhythms (n = 6) calculated from a regression line. A horizontal bar indicates the mean circadian period. Circadian periods of the rhythms from the same SCN are indicated with the same colors. (**f**) Chi-square periodogram demonstrates significantly different circadian periods of *Bmal1* (green) and *Per2* (red) rhythm. A black oblique line in the graph indicates the significance level at p < 0.05. (**g**) Phase difference (ψ) between the circadian *Bmal1* and *Per2* peaks in each cycle. The values are indicated as the mean and SD. (**h**) The mean and SD of ψ in the first and the last 10 cycles is indicated. **p < 0.01 (paired t-test).

In the neonatal SCN, *Bmal1* and *Per2* expression showed robust circadian rhythms (Fig. 1a). The circadian peak on the 1^st^ day of culture was detected at Zeitgeber Time (ZT) 0.8 ± 1.1 (mean ± SD, n = 6) for *Bmal1* and at ZT13.8 ± 1.7 (n = 6) for *Per2*, which were almost in anti-phase, where ZT0 was defined as the time of light-on in the pre-culture light-dark cycle (Fig. 1a–c). The free-running circadian rhythms in culture were not in parallel and much faster in *Bmal1* than in *Per2*, resulting in shortening of the phase difference (ψ) between them (Fig. 1d). The mean free-running circadian period calculated from a regression line fitted to the successive circadian peaks was significantly different between the two rhythms (*Bmal1*, 23.18 ± 0.34 h; *Per2*, 23.41 ± 0.35 h), (Period difference; 0.22 ± 0.05 h, p < 0.01, paired t-test) (Fig. 1e). Chi-square periodogram adapted to the data in the whole cycles (20 cycles) confirmed the difference (*Bmal1*, 23.16 ± 0.34 h; *Per2*, 23.32 ± 0.37 h), (Period difference; 0.16 ± 0.05 h, p < 0.01, paired t-test) (Fig. 1f). The period difference between the *Bmal1* and *Per2* rhythms was not significant in the first 10 cycle (0.20 ± 0.20 h, but significant in the last 10 cycles (0.24 ± 0.12 h, p < 0.05, paired t-test). The lack of significant difference in the first 10 cycles might be due to the lability of circadian rhythms immediately after the perturbation^2^. A small difference in circadian period may result in a large phase-difference between the two rhythms in a long run. Actually, ψ was decreasing in the course of culture, showing 10.17 ± 0.34 h in the first 10 cycles and of 7.66 ± 0.75 h in the last 10 cycles (Fig. 1g). They are significantly different (paired t-test, p < 0.01) (Fig. 1h). These results indicated that internal dissociation occurred between the two circadian rhythms.

**Figure 2:**
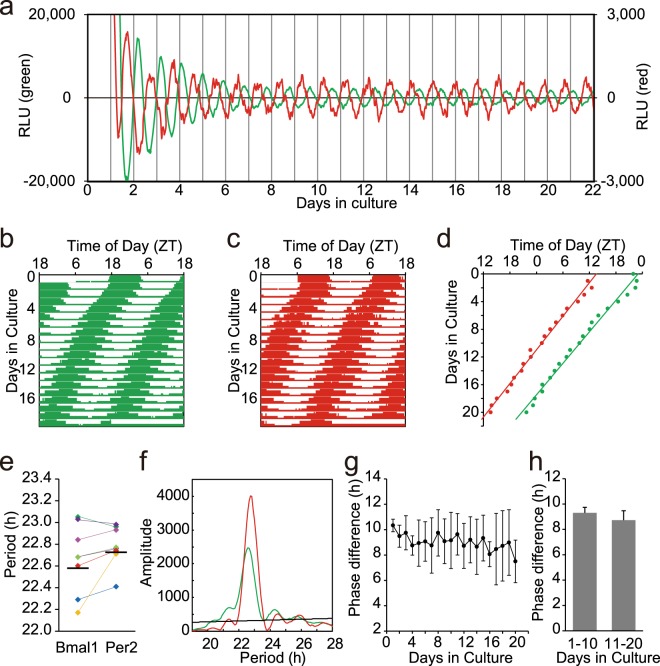
Circadian rhythms of the adult SCN slices of *Bmal1-Eluc:Per2-SLR2* mice (**a**) Sequential records of circadian *Bmal1* (green) and *Per2* (red) rhythm in an adult SCN slice. See also the legend for Fig. 1a. (**b**,**c**) A double plotted circadian *Bmal1* (green) and *Per2* (red) rhythm in a raster format. (**d**) Successive plots of circadian peak phases (*Bmal1*, green; *Per2*, red) and regressions lines fitted to them in the same SCN slice as used in (**a**). (**e**) Circadian periods of *Bmal1* and *Per2* rhythms in the cultured SCN (n = 8) calculated from a regression line. See also the legend for Fig. 1e. (**f**) Chi-square periodogram demonstrates significant but different circadian periods of *Bmal1* (green) and *Per2* (red) rhythm. See also the legend for Fig. 1f. (**g**) A phase difference (ψ) between the circadian *Bmal1* and *Per2* peaks in each cycle. See also the legend for Fig. 1g. (**h**) The mean and SD of ψ in the first and the last 10 cycles is indicated.

In the adult SCN, the *Bmal1* and *Per2* expression showed robust circadian rhythms (Fig. 2a–c) and the antiphasic relation between them persisted at least up to the 20 culture day (Fig. 2d). The mean circadian periods of two rhythms were not significantly different by a regression line (*Bmal1*, 22.58 ± 0.31 h; *Per2*, 22.73 ± 0.18 h, n = 8) (Period difference; 0.12 ± 0.19 h, paired t-test, n.s,) (Fig. 2e) nor by chi-square periodogram (*Bmal1*, 22.64 ± 0.30 h; *Per2*, 22.82 ± 0.14 h, n = 8), (Period difference; 0.18 ± 0.16 h, paired t-test, n.s.). By the latter method, the period difference between the *Bmal1* and *Per2* rhythms was significant in neither the first (0.23 ± 0.41 h, n.s., paired t-test) nor the second 10 cycle (0.20 ± 0.27 h, n.s., paired t-test). The phase-relation was essentially kept constant (Fig. 2g) and the mean ψ did not significantly change during culture (9.31 ± 1.25 h, n.s, for the first 10 cycles and 8.73 ± 1.78 h, n.s, for the last 10 cycles, t-test) (Fig. 2h).

The circadian periods of gene expression rhythms in the adult SCN slice were far shorter than those of the circadian behavior rhythms^23,24^. The circadian periods in the culture system are not necessary the same as those *in vivo*, because the culture conditions are not identical to the tissue environments and the SCN is isolated from the rest of circadian system in the whole body, which abolishes the interactions between them. Similar circadian periods have been reported in the SCN slice culture^24^.

### Phase-dependent phase-shifts of circadian Bmal1 and Per2 rhythms for medium exchange in the neonatal SCN

**Figure 3:**
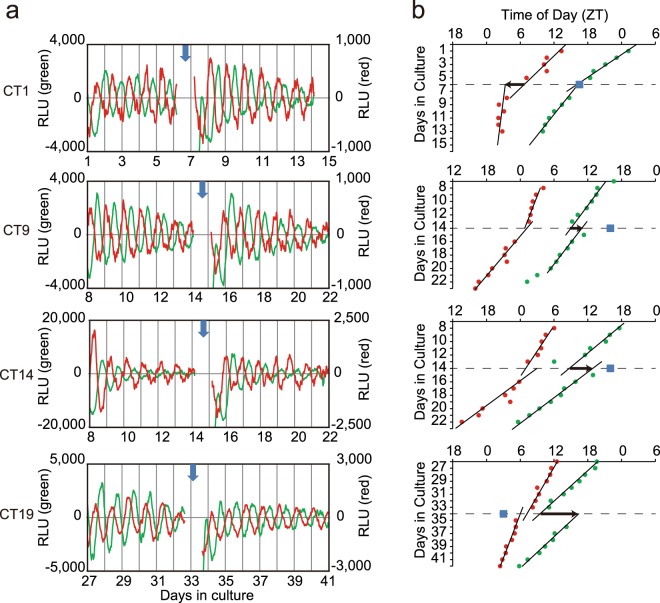
Phase shifts of circadian *Bmal1* and *Per2* rhythm in the neonatal SCN slices in response to medium exchange (**a**) Representative records of phase shifts in response to medium exchange at 4 different phases. The circadian rhythms before and after medium exchange were illustrated for *Bmal1* (green) and *Per2* (red). See also the legend for Fig. 1a. The time of medium exchange was indicated with a vertical arrow. (**b**) Successive circadian peaks with regression lines fitted to them before and after medium exchange in the same SCN as used for (**a**). The abscissa indicates times of day as ZT and the ordinate days in culture. A square column in each panel indicates the time of medium exchange. A horizontal arrow indicates the direction and magnitude of phase-shifts calculated from two regression lines (see text).

Exchange of culture medium with fresh one induced a phase-shift in the circadian *Bmal1* rhythm in the neonatal SCN slice, depending on the circadian phase where medium exchange was done (Fig. 3a). The medium exchange was repeated 3 to 4 times for one SCN slice at intervals of 6–9 cycles and 22 exchanges in total were performed for 6 neonatal SCN. The amount of phase-shift was calculated in such a way that two regression lines were obtained by fitting successive 6–9 circadian peaks before and after medium exchange and a phase difference was obtained by forward and backward extrapolations of the regression lines onto the day of medium exchange (Fig. 3b). The phase-shift was expressed in degrees as well as in hours and the mean phase-shift was calculated in 6 h bins in circadian time (CT), where the circadian peak of *Bmal1* expression was defined as CT1.

**Figure 4:**
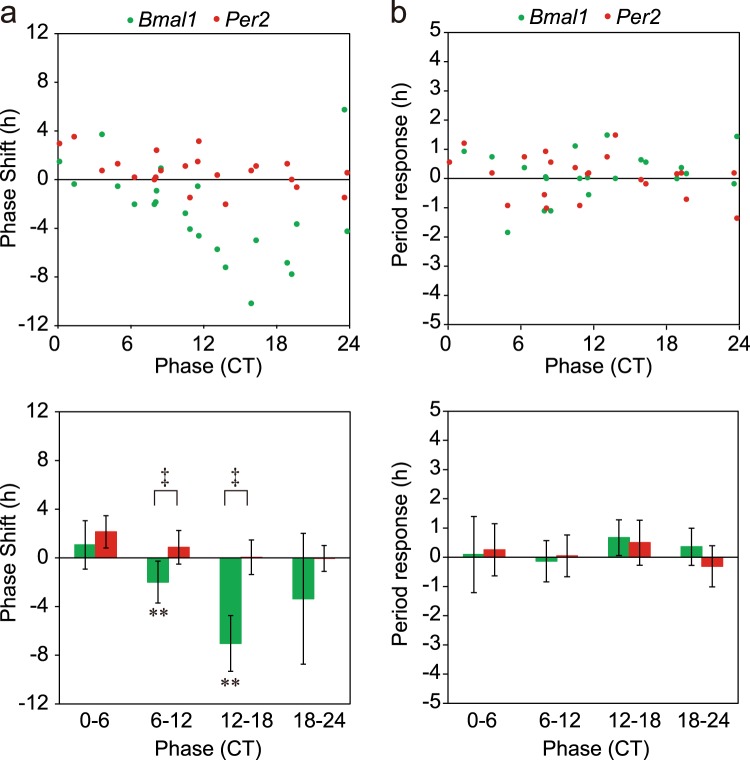
Phase-response curve and period-response curve for medium exchange of circadian *Bmal1* and *Per2* rhythms in the neonatal SCN (**a**) Phase-responses in individual slices (n = 22) of circadian *Bmal1* (green circle) and *Per2* (red circle) rhythms in the neonatal SCN (upper). The abscissa indicates the circadian phase in CT, where CT1 is the peak phase of *Bmal1* rhythm and the ordinate indicates phase-shifts in hours with a positive sign for phase-advance shifts and with a negative for phase-delay. Mean phase-shifts with SD at CT0–CT6 (n = 5), CT6–CT12 (n = 9), CT12–CT18 (n = 4) and CT18–CT20 (n = 5) (lower). A significant phase-shift is indicated with asterisk (*) and a significant difference between the phase-shifts of two circadian rhythms is shown with dagger (^†^). **p < 0.01 (one-way repeated measure ANOVA), ^‡^p < 0.01 (two-way repeated measure ANOVA with post hoc Tukey-Kramer test). (**b**) Period-responses obtained from the same experiment as (**a**). Period-responses in individual slices of circadian *Bmal1* (green circle) and *Per2* (red circle) (upper), and the mean responses with SD at the 4 circadian phases (lower).

Significant phase-delay shifts were detected at CT6-CT12 (n = 9) and CT12-CT18 (n = 4) in the *Bmal1* rhythm (Fig. 4a). Interestingly, a significant phase-advance shift was not observed in the *Bmal1* rhythm. On the other hand, significant phase shifts were not observed in the *Per2* rhythm. Thus, the magnitude of phase-shift was significantly larger in the *Bmal1* rhythm than in the *Per2* at CT6-CT12 and CT12-CT18 (p < 0.01, one-way ANOVA). The phase-dependent phase-shift was also confirmed by the Rayleigh plot (Supplementary Figs 2 and 3).

Changes in the circadian period by medium exchange were plotted in a similar way to that for phase-shifts (Fig. 4b). Phase-dependency of the period change was not detected for either of circadian rhythms.

### Phase-responses of circadian Bmal1 and Per2 rhythm in the adult SCN and effects of TTX treatment

**Figure 5:**
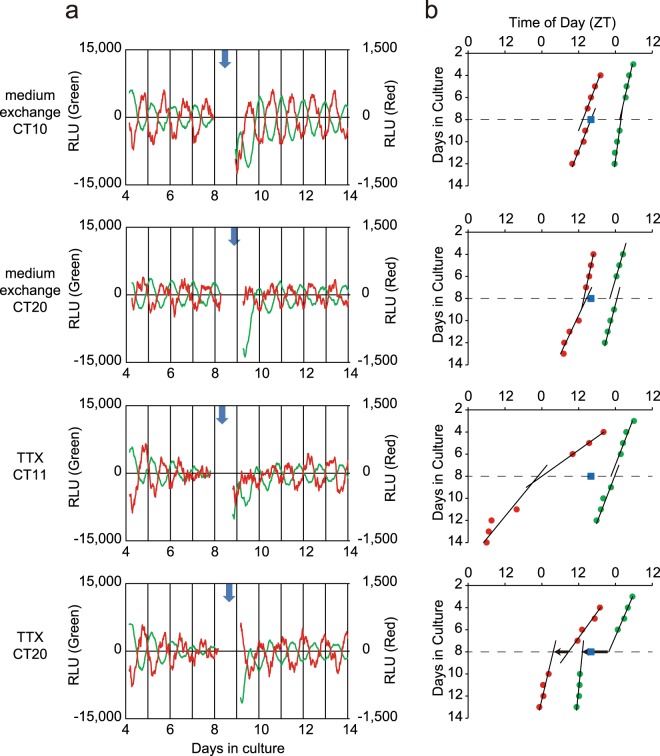
Phase shifts of circadian *Bmal1* and *Per2* rhythm in the adult SCN slices in response to medium exchange and effects of TTX pretreatment (**a**) Representative records of phase shifts in response to medium exchange in the adult SCN pretreated with distilled water (upper, upper most) and with TTX (lower, lower most). See also the legend for Fig. 3a. (**b**) Successive circadian peaks with regression lines fitted to them before and after medium exchange in the same SCN as used for (**a**). See also the legend for Fig. 3b.

The different phase-responses to medium exchange were detected in the adult SCN slice (Fig. 5) from those in the neonatal SCN in both circadian *Bmal1* and *Per2* rhythms. In this experiment, 5.2 μl of distilled water (Gibco, Japan) was added to the dish as the control of following TTX experiment. The SCN slice on a culture membrane was removed from a culture dish. After application of distilled water, culture medium was mixed and the membrane with an SCN slice was returned to the dish. No significant phase-shift was detected by this procedure.

**Figure 6:**
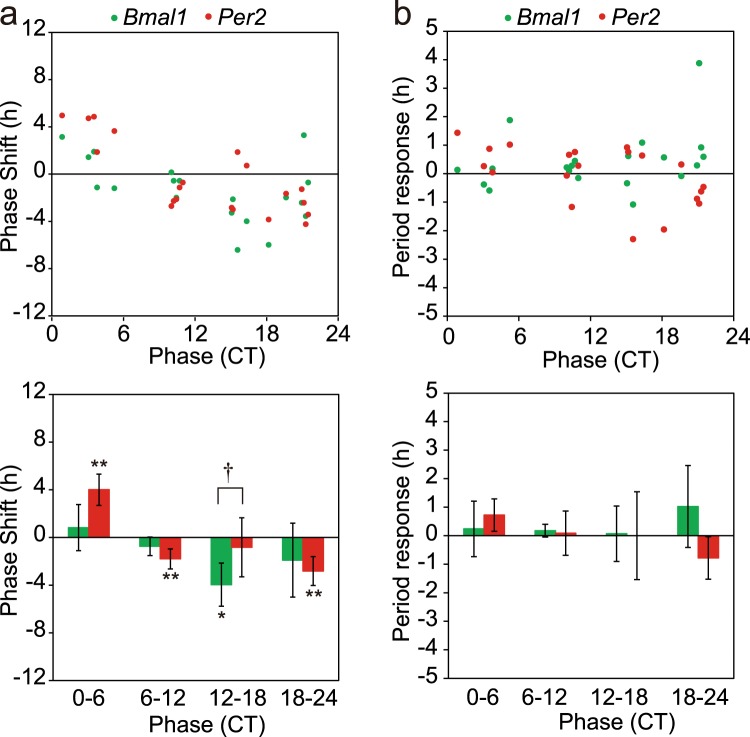
Phase-response curve and period-response curve for medium exchange of circadian *Bmal1* and *Per2* rhythms in the adult SCN (**a**) Phase-responses in individual slices (n = 20) of circadian *Bmal1* (green circle) and *Per2* (red circle) rhythms in the adult SCN (upper). The SCN was pretreated with distilled water (see text). Mean phase-shifts with SD at CT0–CT6 (n = 5), CT6–CT12 (n = 4), CT12–CT18 (n = 5) and CT18–CT20 (n = 6) (lower). See also the legend for Fig. 4a. A significant phase-shift is indicated with asterisk (*). *p < 0.05, **p < 0.01, and a significant difference in the response between the two circadian rhythms is shown with dagger (^†^). ^†^p < 0.05. (**b**) Period-responses obtained from the same experiment as (**a**). Period-responses in individual slices of circadian *Bmal1* (green circle) and *Per2* (red circle) (upper), and the mean responses with SD at the 4 circadian phases (lower).

A significant phase-shift was detected in the *Bmal1* rhythm at CT12-CT18 (n = 5) (Fig. 6a). However, the magnitude of phase-shift was significantly smaller than that in the neonatal SCN (4.0 ± 1.8 h v.s. 7.0 ± 2.3 h; p < 0.01, two-way ANOVA with post-hoc Tukey-Kramer test). The result was confirmed by the Rayleigh plot (Supplementary Fig. 2). On the other hand, not only phase-advance shift at CT0-CT6 (n = 5) but also phase-delay shifts at CT6-CT12 (n = 4) and CT18-CT24 (n = 6) were detected in the circadian *Per2* rhythm (p < 0.01, a paired t-test). A significant difference in the magnitude of phase-shift between the *Bmal1* and *Per2* rhythm was detected at CT0-CT6 and CT12-CT18 by the linear statistics (Fig. 6a) and the Rayleigh plot (Supplementary Figs 2 and 3). In summary, the PRC of *Bmal1* rhythm kept essentially the same phase-responsiveness in the adult SCN as in the neonatal, but the magnitude of phase-shifts was decreased. On the other hand, the PRC developed for the *Per2* rhythm in the adult SCN. The phase of PRC, however, was different from that of *Bmal1* rhythm, showing both the phase-delay and phase-advance portion. Changes in the circadian period by medium exchange were not phase-dependent for either of circadian rhythms (Fig. 6b).

**Figure 7:**
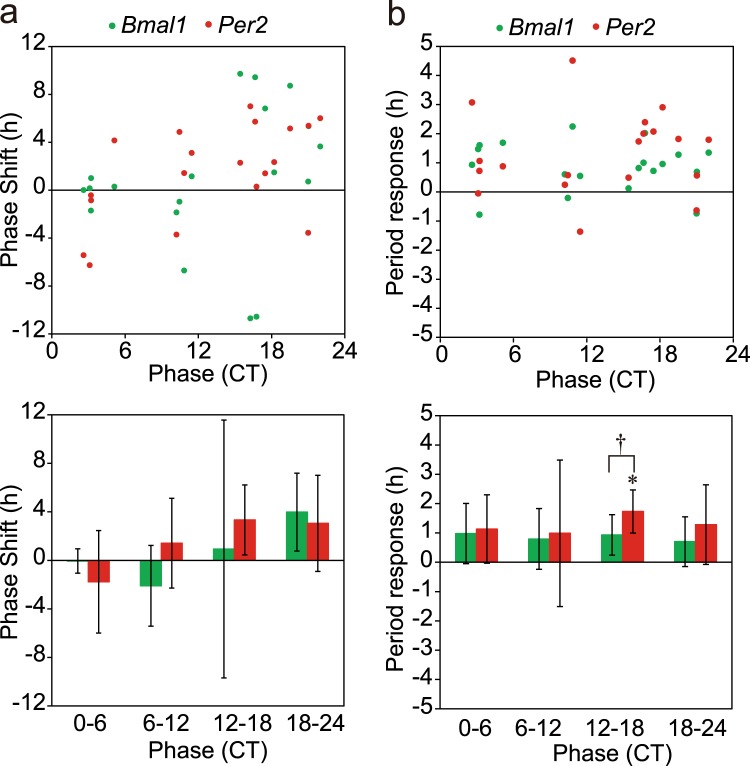
Phase-response curve and period-response curve for medium exchange in the adult SCN pretreated with TTX (**a**). Phase-responses (n = 19) of circadian *Bmal1* (green circle) and *Per2* (red circle) rhythms in the adult SCN (upper panel). The SCN was pretreated with TTX (see text). Mean phase-shifts with SD at CT0–CT6 (n = 5), CT6–CT12 (n = 4), CT12–CT18 (n = 5) and CT18–CT20 (n = 5) (lower panel). See also the legend for Fig. 4a. (**b**) Period-responses obtained from the same experiment as (**a**). Period-responses in individual slices of circadian *Bmal1* (green circle) and *Per2* (red circle) (upper), and the mean responses with SD at the 4 circadian phases (lower). A significant period-response is indicated with asterisk (*) and a significant difference in the response between the two circadian rhythms is shown with dagger (^†^). *p < 0.05, ^†^p < 0.05.

In order to understand a role of SCN neuronal network in phase-shifts induced by medium exhange, tetrodotoxin (TTX), a sodium channel blocker, was added to the culture medium. TTX (Wako, Osaka, Japan) was dissolved in distilled water and 5.2 μl of TTX solution was added to the culture dish (the final concentration, 200 nM) in the same manner as in the control. This dosage of TTX was enough to completely suppress the spontaneous discharge in the SCN slice on a multi-electrode dish^25^. TTX pretreatment itself did not affect the circadian *Bmal1* and *Per2* rhythms. Medium exchange in the TTX pretreated SCN showed different effects on the magnitude of phase-shifts as well as phase responsiveness in both circadian rhythms from those in the control SCN (Fig. 7a). The mean phase-shift in absolute value was significantly larger in the TTX pretreated SCN than in the vehicle treated for both the *Bmal1* and *Per2* rhythms (*Bmal1*: 2.93 ± 1.59 h for vehicle, 5.68 ± 4.45 for TTX, f = 28; *Per2*: 2.94 ± 1.22 h for vehicle, 4.23 ± 1.76 for TTX, f = 32, p < 0.05, t-test). However, TTX pretreatment abolished the phase-dependency of phase-shifts by medium exchange for both circadian rhythms. On the other hand, the Rayleigh plot revealed a significant phase-shifts of the *Bmal1* rhythm at CT12-CT18 (Supplementary Fig. 2), which was almost 180 degrees from the phase before medium exchange.

The circadian period was significantly lengthened by medium exchange for both rhythms (period difference: *Bmal1*, 0.85 ± 0.83 h, p < 0.001; *Per2*, 1.30 ± 1.40 h, p < 0.001, n = 19, paired t-test). And the period change was significantly at CT12-CT18 in the *Per2* rhythms (p < 0.05, t-test).

## Discussions

Simultaneous monitoring of *Bmal1* and *Per2* reporter expression enabled us to analyze dynamics of the interlocked molecular loops for circadian rhythm generation. Although the intensity of bioluminescence emitted from SLR2 was smaller than that from ELuc, the high transmission ration for a long-pass filter (620 nm) and the strong expression of *Per2* gene made a detection of circadian rhythm possible. The circadian rhythms in *Bmal1* and *Per2* expression in the neonatal SCN slices showed internal dissociation during culture. The dissociation was not detected in the adult SCN. TTX pretreatment in the adult SCN enhanced phase-shifts but disorganized the phase-responsiveness in both circadian rhythms. These findings indicate that the circadian rhythms in *Bmal1* and *Per2* expression in the cultured SCN are dissociable and regulated differently by different oscillating mechanisms. There is a developmental change in the circadian organization of the SCN for *Bmal1* and *Per2* expression.

The *Bmal1* loop has been regarded to interlock with the core molecular loop for circadian rhythm generation through activation and suppression of RevErbα/ROR response element in the promoter of *Bmal1* gene^5,6^. Pharmacological manipulation of RevErbα was reported to alter the amplitude of the circadian *Bmal1* and *Per2* expression without changing the circadian period^26^. However, knocking out of RevErbα did not affect the circadian *Per2* expression rhythm at all, making us doubt an essential role of the *Bmal1* loop for circadian rhythm generation^27^. The present study demonstrated that the *Bmal1* and *Per2* expression rhythms in the SCN were dissociable, showing steady changes in the phase-relationship between them. This dissociation is not likely a transient disarrangement of constituent elements of the molecular loops by slice preparation, since it was observed in a long-run culture and gets larger in the later stage than in the immediate after slice preparation (Fig. 1). The dissociation is likely due to distinct oscillations underlying the two circadian rhythms, namely the oscillations generated by the core molecular loop and the *Bmal1* loop. These findings are consistent with our previous result that *Bmal1* and *Per2* transcriptions in the cultured SCN slice of adult mice showed different responses to cycloheximide, an inhibitor of protein synthesis^28^. A similar dissociation of circadian rhythms in different clock gene expression was reported after an abrupt phase-shift of light-dark cycle^29^ or an single light pulse^30^.

It is not known whether they are completely independent of each other or not, since changes of the phase difference did not exceed beyond 12 hours. It is also not known whether the dissociation occurs in the intracellular oscillations or between two different SCN regions where either of the two clock gene expression is predominant. Clock gene expression shows regional specificity in abundance and period^31–33^. The circadian oscillation in the dorsomedial SCN was reported to be faster than that in the ventromedial SCN^33,34^. However, such regional specificity is not known for the *Bmal1* and *Per2* expression rhythms.

Dissociation between the circadian *Bmal1* and *Per2* expression rhythms was also detected in phase-response to medium exchange (Fig. 3). Medium exchange is known to produce phase-dependent phase-shifts in clock gene expression in the cultured SCN^35^. Previously, we reported that the circadian *Bmal1* rhythm in the neonatal SCN slice of mice produced phase-dependent phase shifts in response to medium exchange, whereas the phase shifts was not significant in the adult SCN^19^. The differential responses in the neonatal and adult SCN in the present study are consistent with the previous results. Importantly, the circadian *Per2* rhythm in the neonatal SCN did not significantly respond to medium exchange. As a result, the phase-relation between the circadian *Bmal1* and *Per2* rhythm was changed significantly. Interestingly, the responsiveness of circadian rhythms changed in the adult SCN. The amount of phase-shifts was reduced in the circadian *Bmal1* rhythm, whereas small but significant phase-shifts were detected in the *Per2* expression rhythm. In addition, the shape of PRC of the *Per2* rhythm was different from that of the *Bmal1*.

The circadian period was not systematically affected by medium exchange in either of rhythms, regardless of whether it was in the neonatal or in the adult SCN (Figs 4b and 6b). The finding suggests a different mechanism of phase-shifts from that induced by lights, in which a phase-delay shift was associated with a lengthening of the circadian period and a phase-advance shift with a shortening of the period^36^.

The mechanism of phase-shifts induced by medium exchange is not well understood. Only fresh medium has a potency to produce phase-shifts^19^. TGFβ in the medium was reported critical to induce phase-shifts by medium exchange^37^. On the other hand, the responsiveness of the SCN circadian pacemaker to external perturbation depends substantially on the neural network^38,39^. In this respect, differential responses at different developmental stages and effects of TTX are most interesting. The neural network in the SCN was reported to ontogenetically change^15,17,18^. Actually, the neonatal SCN has less synaptic connections than in the adult^40,41^. TTX blocks neural communication in the SCN and decouples not only among the cellular circadian oscillations^2^ but also between the regional oscillations in the dorsomedial and ventrolateral SCN^3^. TTX pretreatment enhanced the phase-shift by medium exchange but abolished the phase-dependency for both circadian rhythms. In addition, TTX pretreatment lengthened the circadian period after medium exchange (Fig. 7). These findings could be ascribed to the disruption of couplings among constituent cellular or regional oscillators in the SCN^42^.

Physiological significance of the present study lies on the developmental changes in entrainment of the circadian clock. The circadian oscillation in the neonatal SCN is entrained by maternal rhythms up to at least the postnatal day 4 and to the 3^rd^ week of life without light information^10^. Such a non-photic entrainment is not evident^12,43^ or exceptional^44^ in adult animals. The mechanism of non-photic entrainment of the SCN circadian pacemaker is not well understood but could be related with the property of neural network in the SCN^15,18^.

It is concluded that the circadian rhythms in *Bmal1* and *Per2* expression in the cultured SCN are dissociable and show different properties to external perturbation. They are most likely regulated by different circadian oscillators. Developmental changes in the SCN neural network might be involved in the properties of circadian organization in the SCN.

## Methods

### Animals

*Bmal1-ELuc*:*Per2-SLR2* mice of C57BL/6 J background^23^ were used in the present study. They carried dual bioluminescence reporters for *Bmal1* and *Per2* expression. Double heterozygous (Bmal1ELuc/+:Per2SLR/+) mice were backcrossed more than 7 times. They were born and reared in our animal quarters where environmental conditions were controlled (temperature 22 ± 2 °C, humidity 60 ± 5%, 12 h light and 12 h dark with lights-on at 0600-1800). Lights were supplied with fluorescent tubes and the light intensity was *ca*. 100 lx on the surface of the animal cage. The littermates were housed in a polycarbonate cage (182 × 260 × 128 mm, CLEA Japan) with their mother until postnatal day 21 when they were weaned and housed with 2–4 littermates of the same sex. They were provided with commercial chow and tap water *ad libitum*. Animal experiments were performed in accordance with Guidelines for the Care and Use of Laboratory Animals in Hokkaido University with the permission No. 13-0064 from the Committee for Animal Experimentation in the Hokkaido University.

### SCN culture and bioluminescence measurement

Neonatal (6 days old) and adult (2–5 months old) mice of both sexes were euthanized by cervical dislocation and decapitation. Their SCN were cultured as described previously^19^. Briefly, the brain was sampled at around the middle of light phase. A coronal brain slice of 300 μm thick was prepared with Microslicer (Dosaka, Osaka, Japan) including the major part of the SCN. A bilateral SCN was dissected from the slice with a surgical knife and explanted on a culture membrane (Millicell CM, pore size 0.4 μm, Millipore) in a 35 mm petridish. The tissues were cultured with 1.3 ml DMEM (Gibco-Invitrogen) supplemented with 0.1 mM D-Luciferin K salt (Wako), NaHCO_3_ (2.7 mM), HEPES (10 mM), kanamycin (20 μg/ml, Gibco), insulin (5 μg/ml, Sigma-Aldrich), putrescine (100 μM, Sigma-Aldrich), human transferrin (100 μg/ml, Sigma-Aldrich), Progesterone HBC complex (2.3 μg/ml, Sigma-Aldrich) and sodium selenite (30 nM, Gibco). The dishes were placed on a luminometer (AB-2550 Kronos Dio, ATTO, Tokyo, Japan) and bioluminescence from SLR2 and ELuc was measured for 1 min at 20 min intervals alternatively with and without a 620 nm long-pass filter (R62 filter, Hoya, Tokyo, Japan). Unless otherwise stated, the measurement was continued without exchanging the culture medium.

### Separation and calculation of emitted bioluminescence from the dual reporter gene products

Bioluminescence emissions from *Bmal1-ELuc* or *Per2-SLR2* were calculated as reported previously^22^. Briefly, the total emission without filtering and the transmitted emission through a R62 filter were measured using the SCN from each single reporter mouse, i.e. *Bmal1-ELuc* mouse or *Per2-SLR2* mouse. The transmission coefficients were obtained from the transmitted emission divided by the total emission (0.03 for ELuc, 0.55 for SLR2). The emissions from *Bmal1-ELuc* and *Per2-SLR2* were evaluated from the following formula.

$${\rm{F}}0={\rm{G}}+{\rm{R}},\,{\rm{F}}1=0.03{\rm{G}}+0.55{\rm{R}},$$

where F0 is the total emission, F1 is the transmitted emission through R62 filter, and G and R are the respective emissions from *Bmal1-ELuc* and *Per2-SLR2*.

### Medium exchange

Exchange of culture medium was done at different phases of circadian *Bmal1* rhythm. After pre-culturing for several days, culture medium was discarded by suction and fresh medium was added to the dish. Culture dishes were immediately returned to the luminometer. The exchange of culture medium was repeated several times at intervals of 6–10 cycles in the neonatal slices but not in the adult. In total, 22 medium exchanges were done for 6 neonatal SCNs. The order of medium exchange was randomized in terms of circadian phase, so that particular CTs were not examined at the earlier or later times in culture. Actually, there detected no order-effect on the phase-shifts by medium exchange.

### TTX treatment

Tetrodotoxin (TTX, Wako, Osaka, Japan) treatment was performed as described previously^25^. Briefly, TTX was dissolved in distilled water (Gibco) to prepare the 50 μM stock solution. An adult SCN slice (n = 19) on a culture membrane was removed from a culture dish and 5.2 μl of TTX solution was added to the dish (the final concentration, 200 nM). As a control, distilled water was added to an adult SCN (n = 20). After mixing culture medium, the membrane with an SCN slice was returned to the dish. Five days later, medium exchange was done as described above. The same volume of distilled water (vehicle) was applied to the culture medium as the control with the same manner.

### Data analysis

The peak circadian phase and the amount of phase-shift were obtained as described previously^19,45^. A set of original time series data of bioluminescence was smoothed by a 5 point (80 minutes) moving average method and detrended by a 24-hour moving average subtraction method. There was no significant difference in the initial circadian peak phases of *Bmal1* and *Per2* rhythm between the neonatal and adult SCN. To calculate the magnitude of phase-shifts, successive circadian peak phases were plotted against culture days and a linear regression line was fitted to the peak phases for at least 7 cycles immediately before and after medium exchange. A difference was calculated between the two phases on the day of medium exchange predicted by forward and backward extrapolations of a regression line. The phase of medium exchange was expressed in CT by dividing 24 h by the circadian period immediately before the medium exchange. The circadian period was determined from the slope of a regression line and validated by chi-square periodogram (ClockLab, Actimetrics).

### Statistics

Significant phase-shift was evaluated by one way repeated measure ANOVA in a linear statistics and by Hotelling’s paired test (Oriana version 4.01, Kovach Computing Services) in a Rayleigh method. The difference of mean phase-sfhits between the circadian *Bmal1* and *Per2* rhythm was compared with a two-way ANOVA with a post-hoc Tukey-Kramer test. A paired t-test and student t-test were used for comparison between two dependent and independent group means, respectively.

## Electronic supplementary material

Supplementary Information

## References

[1] Welsh DK, Logothetis DE, Meister M, Reppert SM (1995). Individual neurons dissociated from rat suprachiasmatic nucleus express independently phased circadian firing rhythms. Neuron.

[2] Yamaguchi S (2003). Synchronization of cellular clocks in the suprachiasmatic nucleus. Science.

[3] Enoki R (2012). Topological specificity and hierarchical network of the circadian calcium rhythm in the suprachiasmatic nucleus. Proc Natl Acad Sci USA.

[4] Reppert SM, Weaver DR (2002). Coordination of circadian timing in mammals. Nature.

[5] Preitner N (2002). The orphan nuclear receptor REV-ERBalpha controls circadian transcription within the positive limb of the mammalian circadian oscillator. Cell.

[6] Sato TK (2004). A functional genomics strategy reveals Rora as a component of the mammalian circadian clock. Neuron.

[7] Ueda HR (2005). System-level identification of transcriptional circuits underlying mammalian circadian clocks. Nat Genet.

[8] Relógio A (2011). Tuning the mammalian circadian clock: robust synergy of two loops. PLoS Comput Biol.

[9] Reppert SM, Schwartz WJ (1983). Maternal coordination of the fetal biological clock in utero. Science.

[10] Ohta H, Honma S, Abe H, Honma K (2002). Effects of nursing mothers on *rPer1* and *rPer2* circadian expressions in the neonatal rat suprachiasmatic nuclei vary with developmental stage. Eur J Neurosci.

[11] Carmona-Alcocer Vania, Abel John H., Sun Tao C., Petzold Linda R., Doyle Francis J., Simms Carrie L., Herzog Erik D. (2017). Ontogeny of Circadian Rhythms and Synchrony in the Suprachiasmatic Nucleus. The Journal of Neuroscience.

[12] Takahashi K, Deguchi T (1983). Entrainment of the circadian rhythms of blinded infant rats by nursing mothers. Physiol Behav.

[13] Honma S, Honma KI, Shirakawa T, Hiroshige T (1984). Maternal phase setting of fetal circadian oscillation underlying the plasma corticosterone rhythm in rats. Endocrinology.

[14] Sasaki Y, Murakami N, Takahashi K (1984). Critical period for the entrainment of the circadian rhythm in blinded pups by dams. Physiol Behav.

[15] Weaver DR, Rivkees SA, Reppert SM (1992). D1-dopamine receptors activate c-fos expression in the fetal suprachiasmatic nuclei. Proc Natl Acad Sci USA.

[16] Weaver DR, Reppert SM (1995). Definition of the developmental transition from dopaminergic to photic regulation of c-fos gene expression in the rat suprachiasmatic nucleus. Brain Res Mol Brain Res.

[17] Ban Y, Shigeyoshi Y, Okamura H (1997). Development of vasoactive intestinal peptide mRNA rhythm in the rat suprachiasmatic nucleus. J Neurosci.

[18] Ono D, Honma S, Honma K (2013). Cryptochromes are critical for the development of coherent circadian rhythms in the mouse suprachiasmatic nucleus. Nat Commun.

[19] Nishide S, Honma S, Honma K (2008). The circadian pacemaker in the cultured suprachiasmatic nucleus from pup mice is highly sensitive to external perturbation. Eur J Neurosci.

[20] Evans JA, Leise TL, Castanon-Cervantes O, Davidson AJ (2011). Intrinsic regulation of spatiotemporal organization within the suprachiasmatic nucleus. PLoS One.

[21] Tokuda I (2015). Coupling Controls the Synchrony of Clock Cells in Development and Knockouts. Biophys J.

[22] Noguchi T, Ikeda M, Ohmiya Y, Nakajima Y (2008). Simultaneous monitoring of independent gene expression patterns in two types of cocultured fibroblasts with different color-emitting luciferases. BMC Biotechnol.

[23] Noguchi T (2010). Dual-color luciferase mouse directly demonstrates coupled expression of two clock genes. Biochemistry.

[24] Nishide S (2006). New reporter system for Per1 and Bmal1 expressions revealed self-sustained circadian rhythms in peripheral tissues. Genes Cells.

[25] Baba K, Ono D, Honma S, Honma K (2008). A TTX-sensitive local circuit is involved in the expression of PK2 and BDNF circadian rhythms in the mouse suprachiasmatic nucleus. Eur J Neurosci.

[26] Solt LA (2012). Regulation of circadian behaviour and metabolism by synthetic REV-ERB agonists. Nature.

[27] Liu AC (2008). Redundant function of REV-ERBalpha and beta and non-essential role for Bmal1 cycling in transcriptional regulation of intracellular circadian rhythms. PLoS Genet.

[28] Nishide S, Ono D, Yamada Y, Honma S, Honma K (2012). De novo synthesis of PERIOD initiates circadian oscillation in cultured mouse suprachiasmatic nucleus after prolonged inhibition of protein synthesis by cycloheximide. Eur J Neurosci.

[29] Reddy AB, Field MD, Maywood ES, Hastings MH (2002). Differential resynchronisation of circadian clock gene expression within the suprachiasmatic nuclei of mice subjected to experimental jet lag. J Neurosci.

[30] Ono D (2017). Dissociation of Per1 and Bmal1 circadian rhythms in the suprachiasmatic nucleus in parallel with behavioral outputs. Proc Natl Acad Sci USA.

[31] Yan L, Silver R (2004). Resetting the brain clock: time course and localization of mPER1 and mPER2 protein expression in suprachiasmatic nuclei during phase shifts. Eur J Neurosci.

[32] Hamada T, Antle MC, Silver R (2004). Temporal and spatial expression patterns of canonical clock genes and clock-controlled genes in the suprachiasmatic nucleus. Eur J Neurosci.

[33] Myung J (2012). Period coding of Bmal1 oscillators in the suprachiasmatic nucleus. J Neurosci.

[34] Koinuma S (2013). Regional circadian period difference in the suprachiasmatic nucleus of the mammalian circadian center. Eur J Neurosci.

[35] Daan S, Pittendrigh CS (1976). A Functional Analysis of Circadian Pacemakers in Nocturnal Rodents. II. The Variability of Phase Response Curves. J Comp Physiol A.

[36] Vitaterna MH (2006). The mouse Clock mutation reduces circadian pacemaker amplitude and enhances efficacy of resetting stimuli and phase-response curve amplitude. Proc Natl Acad Sci USA.

[37] Kon N (2008). Activation of TGF-beta/activin signalling resets the circadian clock through rapid induction of Dec1 transcripts. Nat Cell Biol.

[38] Bernard S, Gonze D, Cajavec B, Herzel H, Kramer A (2007). Synchronization-induced rhythmicity of circadian oscillators in the suprachiasmatic nucleus. PLoS Comput Biol.

[39] To TL, Henson MA, Herzog ED, Doyle FJ (2007). A molecular model for intercellular synchronization in the mammalian circadian clock. Biophys J.

[40] Lenn NJ, Beebe B, Moore RY (1977). Postnatal development of the suprachiasmatic hypothalamic nucleus of the rat. Cell Tissue Res.

[41] Moore RY, Bernstein ME (1989). Synaptogenesis in the rat suprachiasmatic nucleus demonstrated by electron microscopy and synapsin I immunoreactivity. J Neurosci.

[42] Mieda M (2015). Cellular clocks in AVP neurons of the SCN are critical for interneuronal coupling regulating circadian behavior rhythm. Neuron.

[43] Honma S, Honma K, Hiroshige T (1987). Restricted daily feeding during nursing period resets circadian locomotor rhythm of infant rats. Am J Physiol.

[44] Abe H, Honma S, Honma K (2007). Daily restricted feeding resets the circadian clock in the suprachiasmatic nucleus of CS mice. Am J Physiol Regul Integr Comp Physiol.

[45] Nishide S, Hashimoto K, Nishio T, Honma K, Honma S (2014). Organ-specific development characterizes circadian clock gene Per2 expression in rats. Am J Physiol Regul Integr Comp Physiol.

