# Supplementary material for: Two coupled circadian oscillations regulate Bmal1-ELuc and Per2-SLR2 expression in the mouse suprachiasmatic nucleus
Source: Sci Rep. 2018 Oct 3;8:14765. doi: 10.1038/s41598-018-32516-w (PMC6170461; doi:10.1038/s41598-018-32516-w)
Supplement: Supplementary file 1 — Supplementary Information [file 41598_2018_32516_MOESM1_ESM.pdf]

Supplementary Information

Two coupled circadian oscillations regulate *Bmal1-ELuc* and *Per2-SLR2* expression in the mouse suprachiasmatic nucleus

Shinya Nishide[1, 2], Sato Honma[3] and Ken-ichi Honma[4, *]

1 Department of Physiology, Faculty of Medicine, Hokkaido University

2 Department of Occupational Therapy, School of Rehabilitation Science, Health Science University of Hokkaido

3 Research and Education Center for Brain Science, Hokkaido University

4 Study Group for Monitoring of Brain Functions, Hokkaido University Graduate School of Medicine

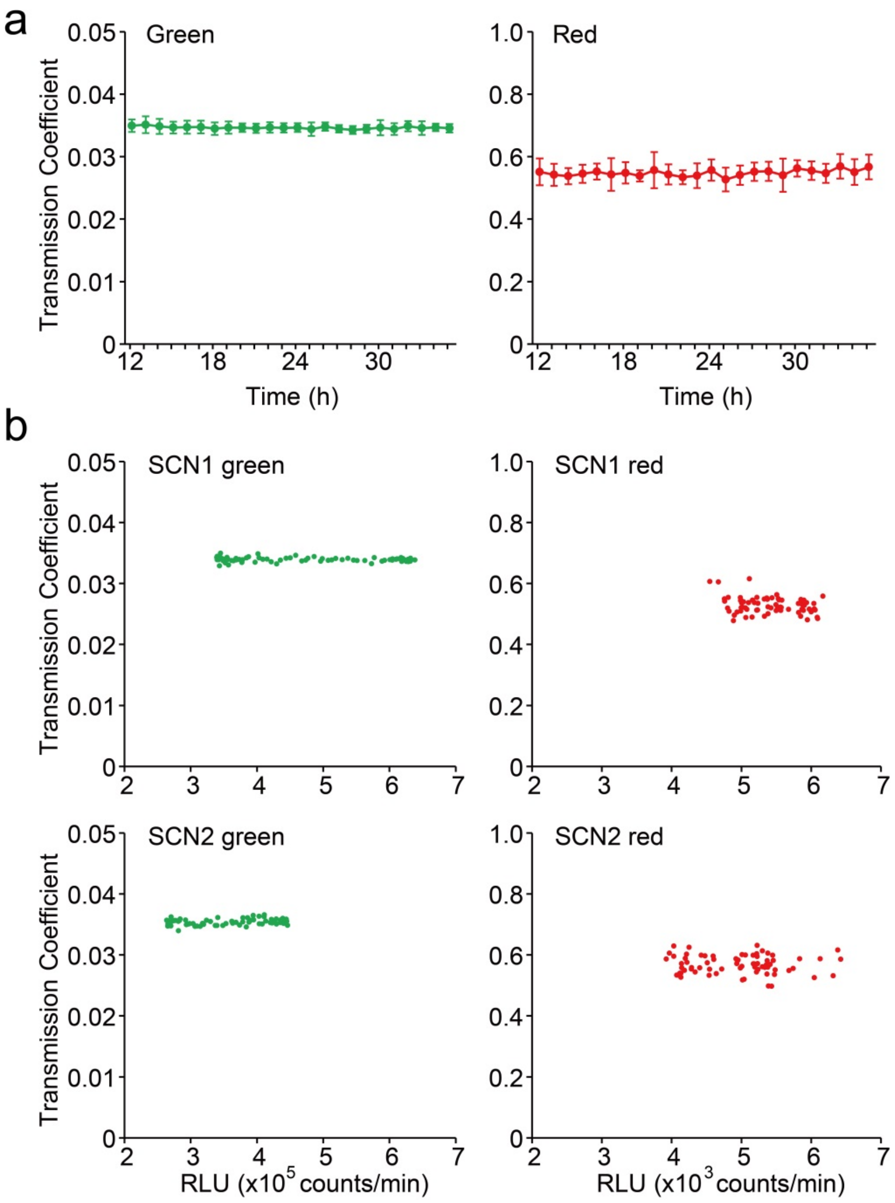

Supplementary Figure 1 Transmission coefficient was essentially independent of the time of day and the intensity of bioluminescence

The transmission coefficient was calculated for *Bmal1-ELuc* and *Per2-SLR2* bioluminescence measured in two SCN slices of mice with a single *Bmal1-ELuc* or *Per2-SLR2* reporter. (*a*) Transmission coefficient in the course of culture for *Bmal1-ELuc* (left) and *Per2-SLR2* (right). Transmission coefficient was kept constant throughout the measurement. (*b*) The transmission coefficient at different intensities of bioluminescence. Transmission coefficient was not significantly different between the low and high bioluminescence intensity in *Bmal1-ELuc* (left) and in *Per2-SLR2* (right), indicating that the transmission coefficient was essentially independent of the intensity of bioluminescence in the range measured in the present study.

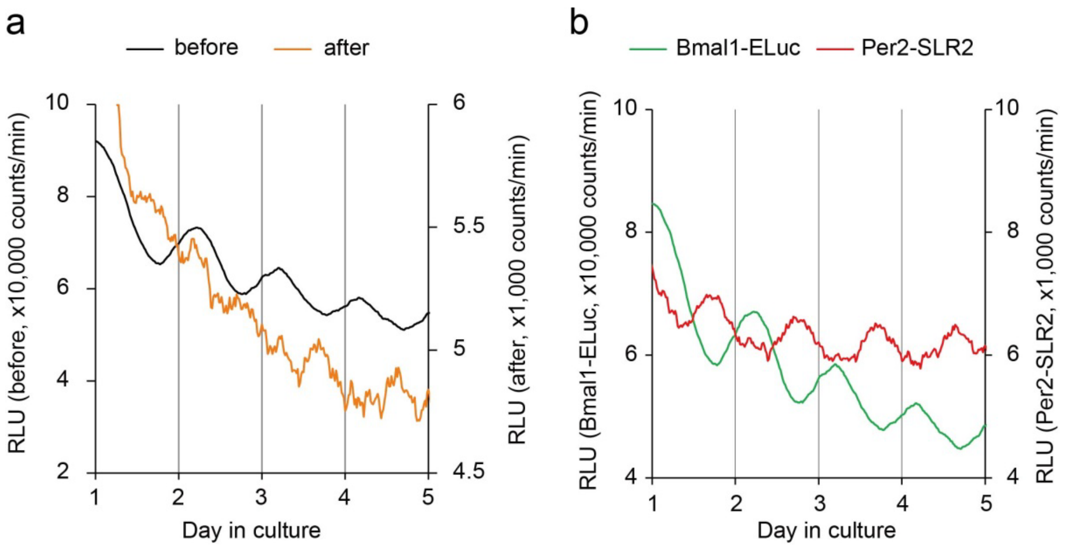

Supplementary Figure 2 Intensity of bioluminescence before and after a long-pass filter application and deduced circadian rhythms in *Bmal1-ELuc* and *Per2-SLR2* expression

(*a*) Bioluminescence before (black) and after (orange) a long-pass filter (620 nm) application for the first 4 culture days. The right ordinate indicates the intensity before filtering and the left after filtering. (*b*) Deduced circadian rhythms of *Bmal1-ELuc* (green) and *Per2-SLR2* (red). See also Materials and Methods in the text.

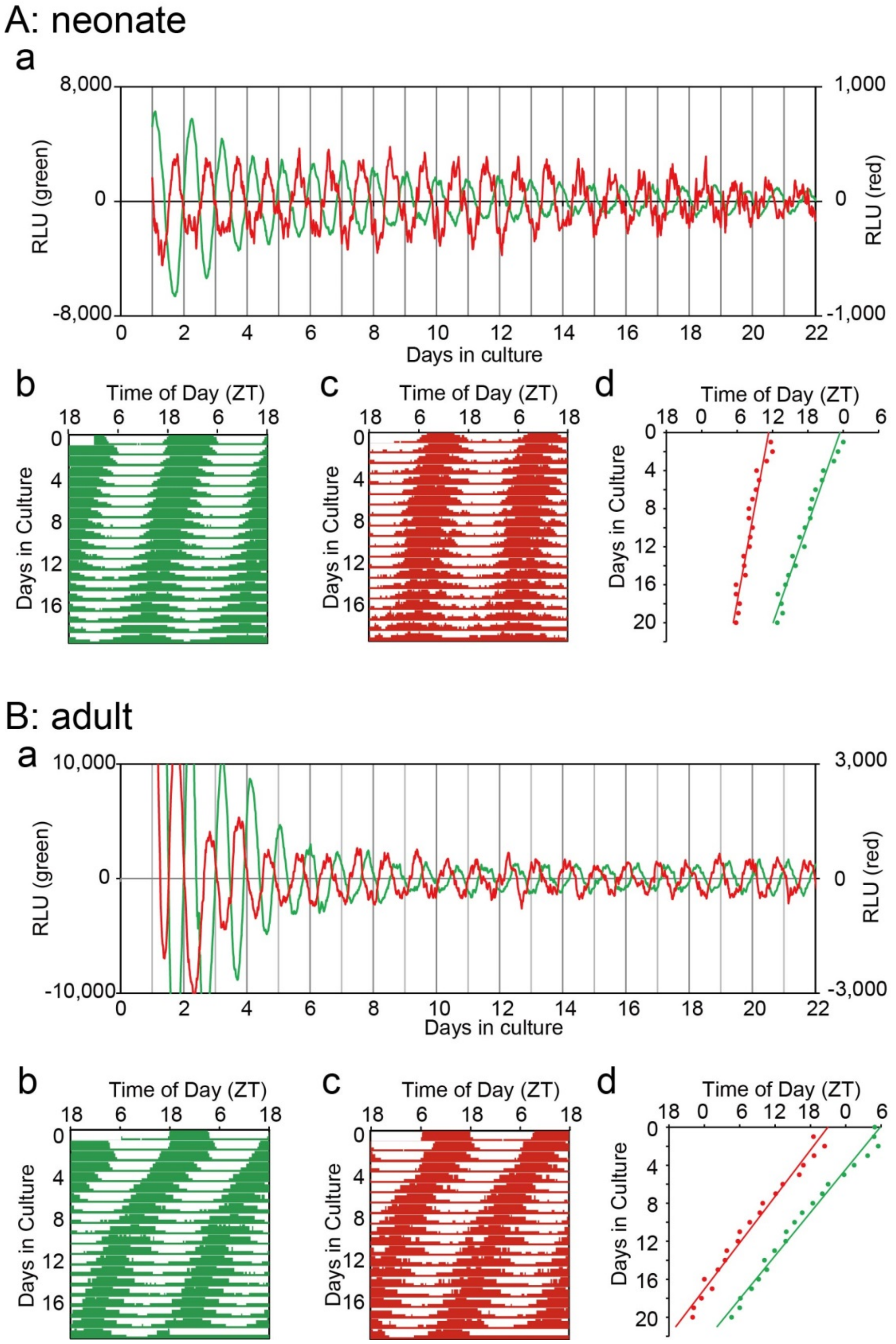

Supplementary Figure 3 Additional examples of circadian rhythms in the neonatal and adult SCN slice of *Bmal1-Eluc:Per2-SLR2* mice

(*a*) Sequential records of circadian *Bmal1* (green) and *Per2* (red) rhythm. (*b*) (*c*) (*d*) A double plotted circadian *Bmal1* (*b*) and *Per2* (*c*) rhythm in a raster format, and successive plots of circadian peak phases (d) (*Bmal1*, green; *Per2*, red) and regressions lines fitted to them in the same SCN slice as used in (*a*). See also the legend of Fig 1.

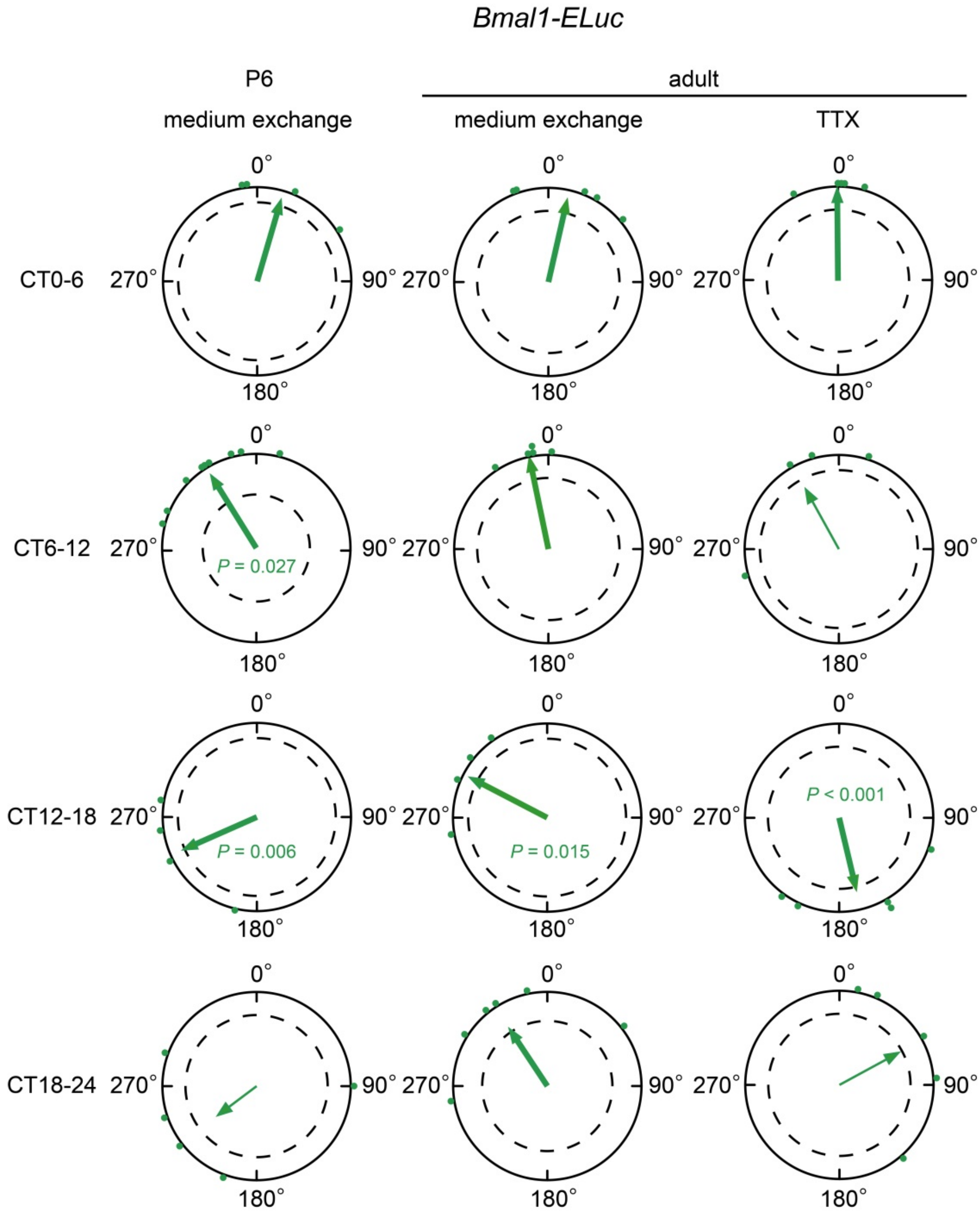

Supplementary Figure 4 Rayleigh plots of circadian peak phases of *Bmal1* rhythm before and after medium exchange

Phase-shifts of the circadian *Bmal1* rhythm in response to medium exchange are illustrated by Rayleigh plot in 4 different circadian phases for the neonatal SCN (left), adult SCN pretreated with distilled water (middle) and with TTX (right). Solid circles indicate the amount of phase shift with degree and broken circles a significance level ($p < 0.05$). Allows indicate the mean phase shift. Direction and length of an arrow indicate the final phase of shift and the variability of phase-shifts, respectively. A thick arrow indicates significant concentration of final phases ($p < 0.05$, Hotelling's paired test) and p-value in the circle significant phase-shift from the initial phase (0°).

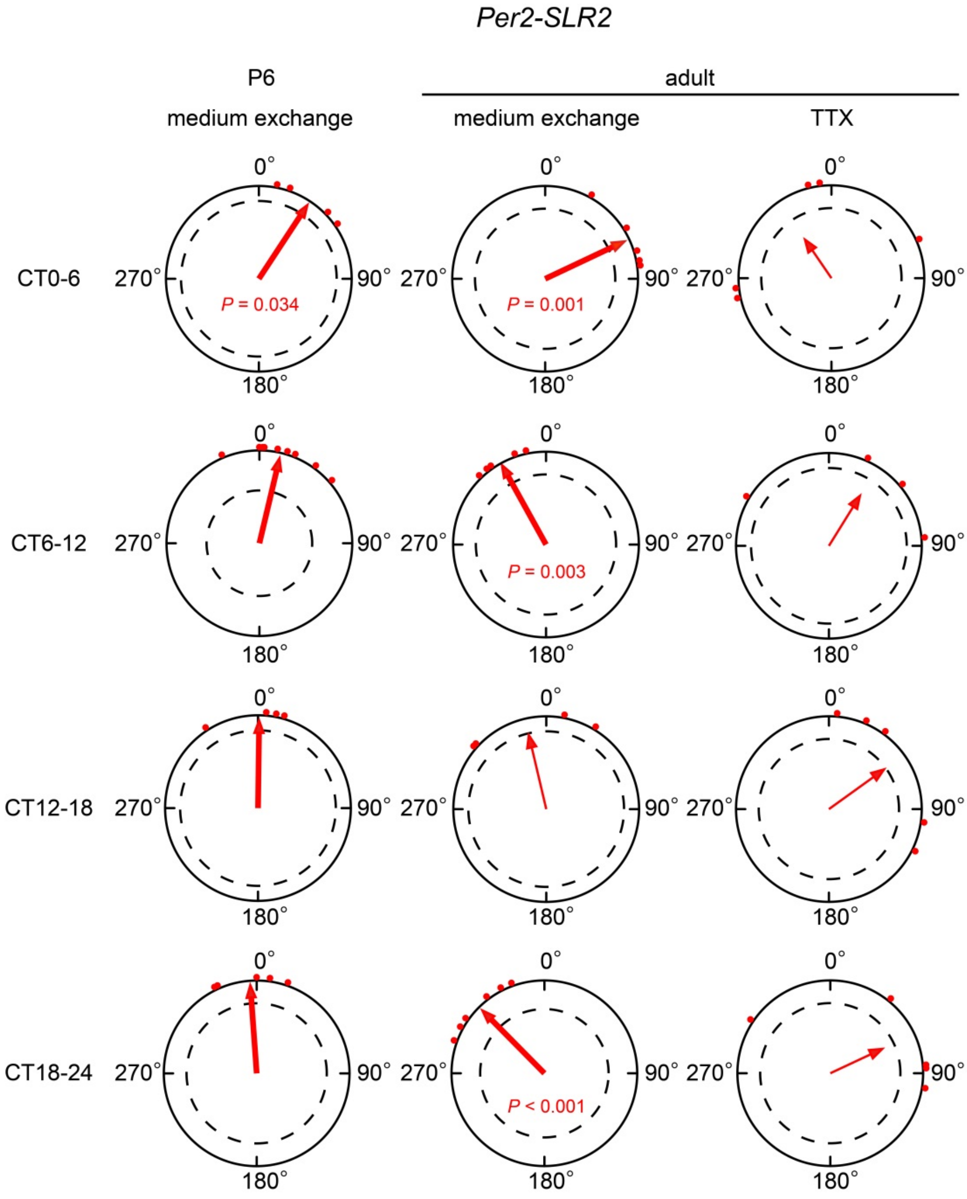

Supplementary Figure 5 Rayleigh plots of circadian peak phases of *Per2* rhythm before and after medium exchange

Phase-shifts of the circadian *Per2* rhythm in response to medium exchange are illustrated by Rayleigh plot in 4 different circadian phases for the neonatal SCN (left), adult SCN pretreated with distilled water (middle) and with TTX (right). See also the legend for Supplementary Fig 1.
